# Validation of Predicted Virulence Factors in *Listeria monocytogenes* Identified Using Comparative Genomics

**DOI:** 10.3390/toxins11090508

**Published:** 2019-08-30

**Authors:** Hossam Abdelhamed, Mark L. Lawrence, Reshma Ramachandran, Attila Karsi

**Affiliations:** Department of Basic Sciences, College of Veterinary Medicine, Mississippi State University, Mississippi State, MS 39762, USA

**Keywords:** *Listeria monocytogenes*, adhesion, invasion, mice infection

## Abstract

*Listeria monocytogenes* is an intracellular facultative pathogen that causes listeriosis, a foodborne zoonotic infection. There are differences in the pathogenic potential of *L. monocytogenes* subtypes and strains. Comparison of the genome sequences among *L. monocytogenes* pathogenic strains EGD-e and F2365 with nonpathogenic *L. innocua* CLIP1182 and *L. monocytogenes* strain HCC23 revealed a set of proteins that were present in pathogenic strains and had no orthologs among the nonpathogenic strains. Among the candidate virulence factors are five proteins: putrescine carbamoyltransferase; InlH/InlC2 family class 1 internalin; phosphotransferase system (PTS) fructose transporter subunit EIIC; putative transketolase; and transcription antiterminator BglG family. To determine if these proteins have a role in adherence and invasion of intestinal epithelial Caco-2 cells and/or contribute to virulence, five mutant strains were constructed. F2365Δ*inlC2*, F2365Δ*eiic*, and F2365Δ*tkt* exhibited a significant (*p* < 0.05) reduction in adhesion to Caco-2 cells compared to parent F2365 strain. The invasion of F2365Δ*aguB*, F2365Δ*inlC2*, and F2365Δ*bglG* decreased significantly (*p* < 0.05) compared with the parent strain. Bacterial loads in mouse liver and spleen infected by F2365 was significantly (*p* < 0.05) higher than it was for F2365Δ*aguB*, F2365Δ*inlC2*, F2365Δ*eiic*, F2365Δ*tkt*, and F2365Δ*bglG* strains. This study demonstrates that *aguB*, *inlC2*, *eiic*, *tkt*, and *bglG* play a role in *L. monocytogenes* pathogenicity.

## 1. Introduction

*Listeria monocytogenes* is a Gram-negative intracellular foodborne pathogen that can cause invasive infection with high mortality rates. Compared to many other foodborne pathogens, *L. monocytogenes* causes a relatively low number of human disease cases, but it is estimated to cause nearly one-fourth of all foodborne-disease-related deaths in the United States each year [1,2]. Pregnant women, newborns, the elderly, and immunocompromised individuals are predominantly affected by an invasive form of illness. Gastrointestinal illness can develop in healthy adults and children as a possible manifestation following ingestion of *Listeria*.

During the infection process, *Listeria* can cross the intestinal, placental, and blood-brain barriers and cause meningoencephalitis, mastitis, abortion, metritis, and septicemia [3,4]. *L. monocytogenes* can grow in a wide range of conditions such as refrigeration (2–4 °C), low pH, and high sodium concentrations [5]. As a result, *L. monocytogenes* has been reported in a wide range of both raw and processed foods, including dairy products, meat products, poultry products, vegetables, and fish products.

The genus *Listeria* currently includes seventeen species [6]. Only the three hemolytic species (*L. monocytogenes*, *L. seeligeri*, and *L. ivanovii*) are considered pathogens [7]. Of these, *L. monocytogenes* is consistently pathogenic and is involved in food-borne outbreaks of listeriosis [8]. Few rare cases of human infections by *L. ivanovii* and *L. seeligeri* were reported [9,10,11]. To search for novel virulence factors of *Listeria*, our group previously performed orthology analysis to identify proteins present in pathogenic strains (EGD-e and F2365) but absent in nonpathogenic strains of the same or closely related species (*L. monocytogenes* strain HCC23 and *L. innocua* strain CLIP1182) [12]. The result revealed a list of 58 secreted proteins, enzymes, transporters, and transcriptional regulators that are uniquely encoded by EGD-e and F2365 compared to HCC23 and CLIP1182. The 58 proteins were classified into different categories according to their functional roles: carbon metabolism, biosynthesis of amino acids, microbial metabolism in diverse environments, cell adhesion, regulation of transcription, biosynthesis of secondary metabolites, capsule synthesis, protein metabolism, signal transduction, and transport. Some of the 58 proteins were previously published and identified to contribute to *Listeria* virulence [12,13,14].

Among the candidate virulence factors, five proteins were selected that were not previously characterized in F2365: putrescine carbamoyltransferase (WP_003724872); InlH/InlC2 family class 1 internalin (WP_003728063); PTS fructose transporter subunit EIIC (WP_003722829); putative transketolase (WP_003725545); and transcription antiterminator BglG family (AAT05528). These five proteins were selected out of 58 proteins to represent different functional groups and classes. Putrescine carbamoyltransferase is one of the four enzymes that constitute agmatine deiminase (AgDI) pathway, which is implicated in bacterial tolerance to acidic environments [15,16]. InlC2 belongs to the internalin protein family, which mediates adhesion and invasion [17,18]. EIIC is one of the phosphotransferase system (PTS) components that contributes to selective transport of sugars across the inner bacterial membrane [19]. Transketolase (TKT) is an enzyme in the non-oxidative branch of pentose phosphate (PP) pathway that connects the PP pathway to glycolysis [20]. *BglG* represents a group of transcriptional antiterminators that control expression of sugar utilization genes [21,22].

Comparative genomics is an emerging field, and it has the capability of predicting putative genes associated with particular phenotypes, including virulence. Comparative genomic analyses of *L. monocytogenes* strain EGD-e with nonpathogenic *L. innocua* strain CLIP1182 revealed potential genetic differences responsible for pathogenicity [23]. Since then, several virulence factors were identified in *L. monocytogenes* by comparative genomics [12,24]. In the current study, we assessed the contribution to virulence of five *L. monocytogenes* genes identified solely by comparative genomics. To assess virulence, we compared the ability of *aguB*, *incl2*, *eiic*, *tkt*, and *bglG* mutants to invade and adhere to intestinal cells, which is an important step to initiate infection and essential for systemic listeriosis. Also, we elucidated the role of the five genes in *L. monocytogenes* virulence using a mice virulence assay. Our findings validate the use of comparative genomics, particularly orthology analysis, as a predictive method to identify putative virulence genes.

## 2. Results

### 2.1. Growth of Mutants

**Figure 1:**
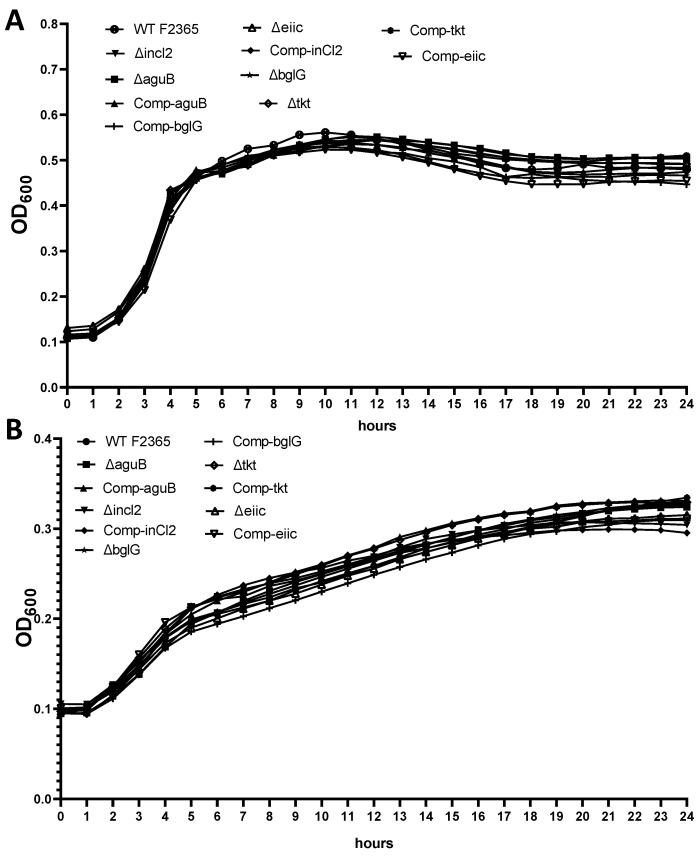
Growth curves for wild-type, mutants, and complement strains in brain heart infusion (BHI) broth (**A**) and minimal medium (**B**) for 24 h at 37 °C. Data represent the mean of three independent experiments, and each was performed in six replicates.

The mutant strains exhibited a similar growth rate to the wild type F2365 strain when grown in brain heart infusion (BHI) as enriched medium or minimal medium (MM) (Figure 1).

### 2.2. Role of aguB, inlC2, eiic, tkt, and bglG in L. Monocytogenes Adhesion

**Figure 2:**
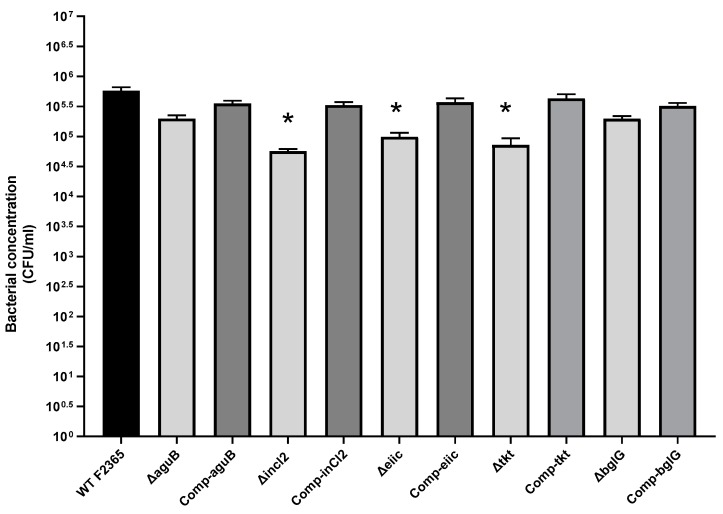
Adhesion of F2365, mutant, and complement strains to Caco-2 cells. Numbers on the Y axis represent bacterial concentration (CFU/mL). Data represent the mean from three independent experiments, and each was performed in four replicates. Error bars reflect standard error from each mean. Asterisks indicate statistical significance between F2365 and mutant strains (*p* < 0.05). Non-significant data were unmarked.

The abilities of wild type F2365 strain and mutant strains to adhere to Caco-2 cells were compared. Caco-2 cells were incubated for 1 h at 37 °C with 10^6^ colony-forming unit (CFU) of bacteria. Strain F2365Δ*inlC2*, F2365Δ*eiic*, and F2365Δ*tkt* showed an approximately 1-log(log_10_), 0.8-log, and 0.9-log reduction in adhesion to Caco-2 cells compared with parent strain F2365. These differences were statistically (*p* < 0.05) significant. F2365Δ*aguB* and F2365Δ*bglG* showed approximately 0.46-log and 0.5-log reduction in adhesion to Caco-2 cells compared to parent strain F2365, and this was not statistically significant. Complementation of the mutations restored adhesion properties (Figure 2).

### 2.3. Role of aguB, inlC2, eiic, tkt, and bglG in L. Monocytogenes Invasion

**Figure 3:**
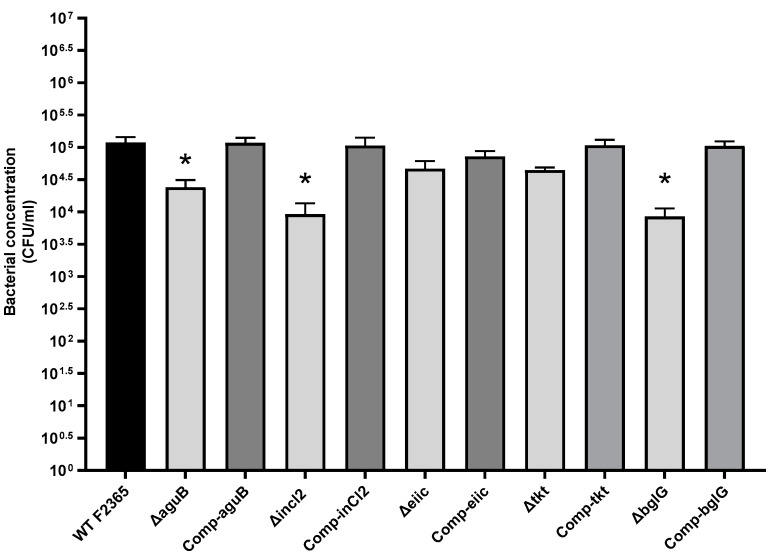
Invasion of Caco-2 cells by F2365, mutant, and complement strains. Numbers on the Y axis represent bacterial concentrations (CFU/mL). Data represent mean colony forming units (CFU) from three independent experiments performed in four biological replicates. Error bars reflect standard error from each mean. Asterisks indicate statistical significance between F2365 and mutant strains (*p* < 0.05).

Invasion of the F2365 strain and mutant strains of *L. monocytogenes* were compared in Caco-2 cells. Caco-2 cells and bacteria were incubated for 1 h at 37 °C followed by an additional 2.5 h in media with gentamicin to kill extracellular bacteria. There were approximately 0.57-log, 0.9-log, and 0.8-log reductions in the invasion in Caco-2 cells infected with F2365Δ*aguB*, F2365Δ*inlC2*, and F2365Δ*bglG*, compared to parent strain F2365, respectively. These differences were statistically significant (*p* < 0.05). F2365Δ*eiic* and F2365Δ*tkt* showed an approximate 0.2-log and 0.4-log reduction in invasion compared to parent strain F2365, and this was not statistically significant (Figure 3). Complementation restored the invasion of the mutant strains to wild type levels.

### 2.4. Virulence and Colonization of Mutant Strains in Mice

**Figure 4:**
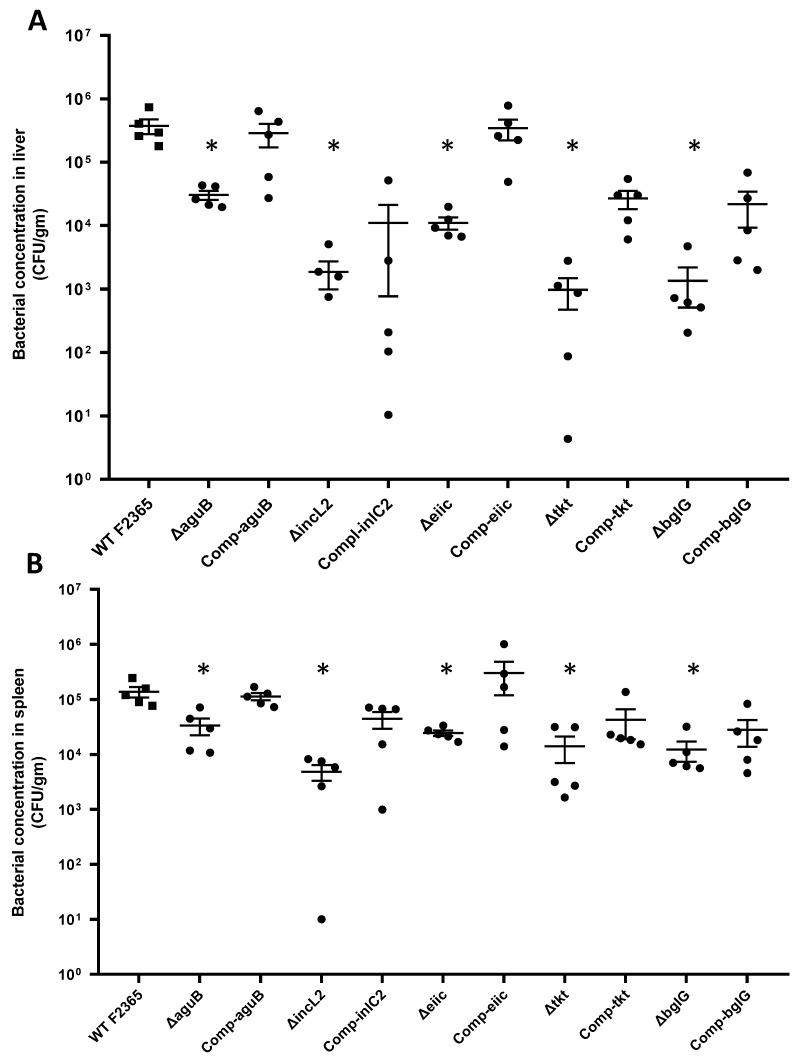
Bacterial concentrations (CFU/gm) in liver (**A**) and spleen (**B**) collected from mice at 3 days post-infection with *L. monocytogenes* F2365, mutant strains, and complement strains. Each dot represents the bacterial concentration in one mouse. The mean ± SE (*n* = 5) numbers of CFU for each strain are indicated by horizontal lines. Statistical analysis was performed by a non-parametric Mann-Whitney test. *, *p* < 0.05.

Virulence of the five mutant strains was assessed in vivo using BALB/c mice. Mice were infected intraperitoneally with 5 × 10^4^ CFU/mL, and bacterial loads in liver and spleen were enumerated 3 days post-infection. Bacteria concentrations in the liver were significantly (*p* < 0.05) higher in mice infected with F2365 strain than mice infected with mutant strains. Bacteria concentrations were reduced about 1-log, 2.3-log, 1.5-log, 2.6-log, and 2.4-log in F2365Δ*aguB*, F2365Δ*inlC2*, F2365Δ*eiic*, F2365Δ*tkt*, and F2365Δ*bglG* compared with the wild-type strain, respectively (Figure 4A). Also, bacterial loads were found to be significantly higher in case of *aguB*, *eiic*, *tkt*, and *bglG* complemented strain as compared to corresponding mutant (*p* = 0.031, 0.0079, 0.0079, and 0.031, respectively), confirming that it was the deletion of the gene which led to the consequent alterations in the bacterial loads. No significant differences between the wild-type and complement strains were observed in these four strains (*p* > 0.05). However, bacterial loads in mice infected with *inlC2* complemented strain were not significantly (*p* = 0.39) different compared to deletion strain. This could be due to a defect in the expression of the *inlC2* during replication of the complemented strain in liver tissue. Moreover, genetic complementation could differ from the native level of expression [25]. Colonization of strain F2365 and complemented strains in the spleen was significantly (*p* < 0.05) higher than the tested mutants. Compared to parent strain F2365, the reduction in bacterial concentrations in spleen were 0.6-log, 1.5-log, 0.7-log, 1-log, and 1-log in F2365Δ*aguB*, F2365Δ*inlC2*, F2365Δ*eiic*, F*2365*Δ*tkt*, and F2365Δ*bglG*, respectively (Figure 4B). 

## 3. Discussion

*L. monocytogenes* is a facultative intracellular bacterium able to penetrate and replicate within mammalian cells [26]. Serotype 4b strain F2365 was isolated from one of the worst bacterial foodborne outbreaks reported in the United States; it was involved in an outbreak of listeriosis in California in 1985 [27]. *L. monocytogenes* pathogenesis includes multiple stages such as internalization, vacuolar escape, intracellular replication, movement by actin mobilization, and cell-to-cell spread [28]. Several *L. monocytogenes* genes whose products are required for these processes have been identified [29]. The aim of the present study was to determine the predictive value of comparative genomics by investigating roles of five previously uncharacterized proteins in *L. monocytogenes* virulence*. L. monocytogenes* ability to adhere and invade phagocytic and non-phagocytic cells is an important aspect of disease pathogenesis. Thus, the intestinal epithelium Caco-2 cell line was used as an in vitro model to evaluate the effects of gene deletion on adhesion and invasion of *L. monocytogenes*. Intraperitoneal inoculation was chosen for this study as it allows *Listeria* to directly disseminate to the systemic sites via the lymphatic system and blood, bypassing the need for adhesion and invasion of the intestine as in oral infection [30]. To evaluate the level of systematic infection, bacteria loads in the liver and spleen were assayed 72 hours after infection. Liver and spleen play an important role in pathogenesis of listeriosis [31]. Shortly after infection, *Listeria* rapidly disseminates to the spleen and liver due to quick uptake by dendritic and Kupffer cells [32]. The majority of the invading bacteria accumulate in the liver and spleen and bacterial replication occurs in the liver and spleen during early stages of infection [33]. Peak burden of *L. monocytogenes* multiplication occurs at 48 to 72 h after infection and mice start to die shortly after this period [34].

In the present study, F2365Δ*aguB* showed approximately 0.57-log reduction in invasion in Caco-2 cells compared to parent F2365 strain. Also, deletion of *aguB* resulted in reduced colonization in liver and spleen as compared to wild-type. The *aguB* gene encoding putrescine carbamoyltransferase mediates phosphorolysis of *N*-carbamoylputrescine to produce putrescine and carbamoylphosphate [15]. This reaction represents the second step of the agmatine deiminase (AgDI) pathway, which includes four genes: An agmatine–putrescine antiporter (*aguD*); agmatine deiminase (*aguA*); putrescine carbamoyltransferase (*aguB*); and a carbamate kinase (*aguC*) [35]. The AgDI pathway plays an important role in the control of cytoplasmic pH and generates metabolic energy in the form of ATP [36,37] by converting agmatine into putrescine, ATP, ammonia, and carbon dioxide [38]. In a previous study, *L. monocytogenes* EGD-e mutants with deletion in Δ*arcA* and Δ*argR* (arginine deiminase ADI pathway genes, which closely resembles AgDI pathway) showed a 10-fold reduction in survival in spleens compared with the wild-type strain in mice following intraperitoneal infection [39]. In *Salmonella* Typhimurium, putrescine plays a critical role in controlling virulence through stimulating the expression of essential virulence loci. Furthermore, a *S.* Typhimurium putrescine mutant displayed defective invasion and survival/replication in epithelial cells and was attenuated in the mouse model compared to the wild-type [40].

In the present study, a Δ*inlC2* mutant was defective in adhesion and invasion to epithelial cells and also showed significant attenuation in the mouse model. These results suggest that *inlC2* contributes to *L. monocytogenes* virulence. Complementation of *inlC2* mutant strain restored the adhesion and invasion to epithelial cells. However, full restoration of wild type behavior was not achieved by *inlC2* complemented strain in liver tissue. This could be due to defect in the expression of the *inlC2* during replication of complement strain in liver. InlC2 is in the internalin family, a diverse group of proteins required for entry of *L. monocytogenes* into non-professional phagocytic cells, such as epithelial cells, hepatocytes, fibroblasts, or endothelial cells [41]. The internalin family is characterized by tandem arrays of leucine rich repeats (LRR) [18]. *inlC2* encodes a secreted protein of 548 aa and is in the same operon with *inlD* and *inlE*. The secreted InlC2 protein plays a significant role in *L. monocytogenes* cell to cell spread [42]. Its amino acid sequence is highly homologous to InlH, with only 13 amino acid differences. Both InlC2 and InlH have the same LRR domain and C-terminal regions, and both are regulated by the same stress-responsive sigma factor σ [43,44]. In some *L. monocytogenes* strains such as EGD and 10403S, the 5’end of *inlC2* is fused with the 3′ end of *inlD* to result in *inlH* [45,46]. InlC2 upregulates antigens during infection and thus may be important in *L. monocytogenes* pathogenesis [47]. Inactivation of *inlH* (which removed *inlD* and *inlC2*) by an in-frame deletion led to attenuated *L. monocytogenes* EGD virulence, evidenced by significantly reduced numbers of bacteria in both liver and spleen compared to the wild-type strain after oral infection of mice [14]. In contrast, no effect on *L. monocytogenes* EGD virulence could be established upon inactivation of *inlC2* by comparing bacterial concentrations in liver and spleen following intravenous injection [45]. In another study, deletion of *inlC2* in *L. monocytogenes* strain M5 significantly enhanced the adherence and invasion to Hela cells, and virulence in murine mouse model. The authors speculate that increased invasion of Δ*inlC2* mutant might be due to elevated production of InlA [48]. This discrepancy between the results may be due to strain differences that lead to difference in protein structure and function or may be due to differences in the cell line type [49,50]. Antibodies to InlC2 are generated in various animal hosts infected with *L. monocytogenes*, indicating that InlC2 may be induced in vivo [51].

Phosphotransferase component EIIC is an essential component of a phosphotransferase system (PTS) that is responsible for recognition, selective binding, and transport of specific sugar molecules through the cell membrane into the cytoplasm [52]. EIIC components are divided into several families, including subclasses for glucose, sucrose, mannitol, fructose, lactose, mannose, and cellobiose [53]. Understanding the role of EIIC (LMOf2365_0661) is of great importance because growth of *L. monocytogenes* as an intracellular pathogen depends on efficient use of sugar from the host. In the present study, F2365Δ*eiic* exhibited a significant reduction in adhesion to epithelial cells. Moreover, *F2365Δeiic* had less bacterial counts in vivo compared to its parent strain. In a previous study, mutation of a PTS EIIC homolog did not impair virulence of *Klebsiella pneumoniae* in the mouse respiratory infection model [54]. Various PTS systems have been associated with *Streptococcus gordonii* adhesion and biofilm formation [55]. Also, an in vivo expression technology screen performed with *K. pneumoniae* strain CG43 indicated that *ptfA*, encoding a fructose phosphotransferase, was positively expressed during BALB/c mice infection [56].

Our study showed that deletion of *tkt*, encoding transketolase enzyme, in F2365 significantly decreased adhesion but did not affect invasion of Caco-2 cells compared to the parent strain. The *tkt* mutation also reduced virulence of *L. monocytogenes* in mice. Transketolase (*tkt*) catalyze the reversible transfer of a ketol group between several donor and acceptor substrates [57,58], thus creating a reversible link between glycolysis and the PP pathway. TKT is responsible for the production of essential cell constituents, such as aromatic amino acids, aromatic vitamins, NADPH, several sugar phosphate intermediates, and pyridoxine [59]. In *Leishmania mexicana*, *tkt* was found to be essential in establishing mammalian cell infection, and *tkt*-deleted *L. mexicana* did not cause any obvious lesions in mice even after 9 weeks [60]. *Moreover*, *tkt1* is induced in vivo in chicken livers and spleens following avian pathogenic *Escherichia coli* (APEC) infection, demonstrating the importance of *tkt* in APEC pathogenesis [61]. Genes *tktA* and *tktB* are essential for APEC survival in chickens [62,63]. Moreover, *tkt*-deleted *Mycobacterium tuberculosis* displayed reduced virulence and intracellular growth in macrophages compared to wild-type strain [64]. In *E. coli*, deletion of *tktA* increased antibiotic and oxidative stress susceptibilities through interaction with *marRAB* operon [65].

In the present study, strains Δ*eiic* and Δ*tkt* adhered to Caco-2 cells less efficiently than wild type strain but the difference in the intracellular multiplication was not significant. This may be due to the fact that the role of these two genes is limited to entry process and contact between *L. monocytogenes* and host cells. Similar to this finding, the deletion of *L. monocytogenes* either *fliF* or *fliI* abolishes flagella assembly and impairs the bacterial adhesion to nonphagocytic cells. However, intracellular multiplication of null mutants is apparently not affected [66]. In the same study, the authors monitored the adhesion and intracellular multiplication of less motile *L. monocytogenes* strains. The result demonstrated that all the strains adhered less efficiently (between 2 and 10 times less) than EGD-e to Caco-2 cells but the intracellular survival was not affected in any of the isolates, suggesting that expression of these genes might play a role in the adhesion to epithelial cells but has no impact on intracytosolic multiplication [66].

The present work demonstrated that mutation of *bglG* impaired bacterial invasion severely but not adhesion. Furthermore, *bglG* deletion reduced the virulence of *L. monocytogenes* in mice. BglG is an RNA-binding transcriptional antiterminator that regulates expression of phosphoenolpyruvate phosphate transferase systems (PEP-PTS) by preventing premature termination of the transcription process [67]. In *E. coli*, *bglG* control expression of β-glucoside utilization (*bgl*) operon by binding to *bgl* mRNA at sites that stabilizing and alleviating the formation of the terminator structure and allowing transcription of downstream genes [68,69]. It is more likely that *bglG* is involved in sensing and consumption of carbohydrates from the host cell during infection, thus facilitating pathogenesis [70]. A previous study identified 15 putative *bglG* transcriptional antiterminators in the *L. monocytogenes* chromosome, all of which appear to be located either up or downstream from genes encoding PTS system [71]. Deletion mutants in lmo0402 and lmo0501 genes, which encode a *bglG* transcriptional antiterminator, exhibited they are essential for attachment of *L. monocytogenes* EGD-e strain at lower temperatures [72].

The in vivo study was performed to determine bacterial count in liver and spleen following dissemination of bacteria in the internal organs. However, the health-related parameters such as body temperature and weight loss were not recorded during the animal study. Even though body temperature and weight loss are often considered as benchmarks of wellbeing in animal models of acute and chronic disease, measurement of these parameters require handling of animals, which could cause distress and immune suppression that can confound the data and thereby potentially lead to erroneous conclusions [73]. Moreover, body temperature is influenced by numerous factors, including the time of day, the presence and type of bedding, and the number of cage mates [74,75]. Weight loss is a complex factor and does not always indicate the severity of illness or the likelihood of imminent death [76,77].

This study highlights the significant contributions of five genes in the pathogenic potential of *L. monocytogenes* by whether influencing the adhesion (*inlC2*, *eiic*, and *tkt*), invasion (*aguB*, *inlC2*, and *bglG*), and virulence (*aguB*, *inlC2*, *eiic*, *tkt*, and *bglG*) in mice. These results correlated with our comparative genomics and orthology analysis and support the role of such comprehensive and predictive methods in identifying putative and undiscovered virulence factors. However, the mechanisms leading to attenuated virulence in these mutants are not yet completely clear. Future work will focus on further identifying mechanism of attenuation on these strains.

## 4. Material and Methods

### 4.1. Ethics Statement

All animal experiment presented in this study was approved by the Animal Care Committee of the Mississippi State University (protocol # 17-105). Approval date: 11 March 2017.

### 4.2. Bacterial Strains and Growth Conditions

All of the strains and plasmids used in this study are listed in Table 1. Brain heart infusion (BHI) (Difco Laboratories, Detroit, MI, USA) and Luria–Bertani (LB) (Difco Laboratories) broth and agar were used to grow *L. monocytogenes* and *Escherichia coli* strains, respectively. *L. monocytogenes* and *E. coli* were incubated at 37 °C. Strains carrying plasmids were grown in the presence of the following antibiotics: ampicillin (100 μg/mL) for *E. coli* strains, erythromycin (10 μg/mL) and chloramphenicol (10 μg/mL) for *L. monocytogenes* strains. Anhydrotetracycline (ATc, 1.5 μg/mL) was used for inducing the expression of antisense *secY* RNA in the pHoss1 plasmid as described below [78]. Human enterocyte-like Caco-2 (TIB37, ATCC) cell lines were grown in Dulbecco’s Modified Eagle’s Medium (DMEM) (ATCC, Gibco, Manassas, VA, USA) supplemented with 20% fetal bovine serum (Atlanta Biologicals, Norcross, GA, USA) and 1% glutamine incubated at 37 °C with 5% CO_2_ under humidified conditions.

### 4.3. Construction of L. Monocytogenes Mutants

Five genes (Table 2) were targeted for in-frame deletion using a previously published mutagenesis gene excision method [78]. A generic map and more details for the strategy used for genes deletion are present in our previously published manuscript [78]. The sequence of primers used for construction and validation of the mutants are listed in Table 3. Briefly, approximately 1 kb fragment of the upstream and the downstream region of each gene was amplified by PCR using genomic DNA from *L. monocytogenes* strain F2365 as a template. Overlap PCR reactions were used to generate in-frame deletion DNA fragments [81]. These fragments were then cloned into the digested pHoss1 vector and transformed into chemically competent *E. coli* DH5α. After sequencing, the resulting plasmids were electroporated into *L. monocytogenes* strain F2365 and selected in BHI plus erythromycin at 30 °C for 3 days. A single colony was streaked onto BHI without erythromycin and incubated at the nonpermissive temperature (42 °C) for two days to allow the plasmid to integrate into the chromosome. This process was repeated three times. Single colonies were then passed three times on BHI broth at 30 °C before spreading BHI agar containing ATc. The induction of *secY* antisense RNA expression in the presence of ATc inhibits the growth of bacteria that retain the integrated plasmid and allow positive selection for chromosomal excision and loss of the plasmid [82,83]. The presence of mutation in the erythromycin-sensitive clones were verified by colony PCR using the primer pair A and D. Final confirmation of the deletion was confirmed by sanger sequencing. The constructed mutants were designated F2365Δ*aguB*, F2365Δ*inlC2*, F2365Δ*eiic*, F2365Δ*tkt*, and F2365Δ*bglG*.

### 4.4. Complementation of the Mutant Strains

Complementation procedures were performed by inserting a copy of the wild-type gene with its promoter region into the appropriate mutant strains using the pPL2 site-specific shuttle integration vector [80]. The integration site of pPL2 have no observed effect on virulence phenotypes of *Listeria* [80]. Briefly, coding fragments of *aguB*, *inlC2*, *eiic*, *tkt*, and *bglG* were amplified by PCR using primers listed in Table 3. PCR products were digested with *Sac*I and *Sal*I enzymes, ligated into pPL2, and transformed into *E. coli* DH5α. To verify the correct assembly of the plasmids, colony PCR and sequencing were performed. The resulting plasmids were then electroporated into *L. monocytogenes* mutant strains and selected on BHI agar plates containing chloramphenicol. Chromosomal insertion of *aguB*, *inlC2*, *eiic*, *tkt*, and *bglG* was confirmed by PCR. Complemented strains generated through this approach were designated F2365Δ*aguB*::*aguB*, F2365Δ*inlC2*::*inlC2*, F2365Δ*eiic*::*eiic*, F2365Δ*tkt*::*tkt*, and F2365Δ*bglG*::*bglG*.

### 4.5. Growth Kinetics of Mutants

Growth F2365 wild-type strain, mutants, and complemented strains in BHI broth and Minimal Medium (MM) [84] at 37 °C were compared. Growth assays were achieved in a 96-well plate. Overnight cultures of each strain in BHI were adjusted to an optical density at λ = 600 nm (OD600) of 1.00 and diluted 1:50 into BHI or MM. The plates were incubated in a Multi-Mode Reader (BioTek Cytation 5) at 37 °C for 24 h. OD600 was measured every hour. All growth experiments were performed in three independent experiments, and each experiment was run with six replicates.

### 4.6. Adherence and Invasion Assay

Adherence of *L. monocytogenes* F2365 wild-type, mutant, and complemented strains to Caco-2 epithelial cells was evaluated as described [85]. In brief, Caco-2 cells were seeded into 24-well tissue culture plates at 10^5^ cells per well. The cells were infected with bacteria (10^6^ CFU) at a multiplicity of infection (MOI) of 10 bacteria per cell and incubated at 37 °C for 1 h in the presence of 5% CO_2,_ after which the medium was removed. Infected monolayer cells were then washed three times with phosphate-buffered saline (PBS) to eliminate non-adherent bacteria and lysed with 0.5% Triton X-100. Appropriate serial dilutions were prepared in PBS and spread on BHI agar for enumeration of bacterial cells. Adherence assay were performed in three independent experiments with four biological replicates for each infection.

*L. monocytogenes* F2365, mutant, and complemented strains were tested for their ability to invade Caco-2 cells as described previously [86]. Bacteria were added to the Caco-2 cell monolayer to yield MOI of 10 bacteria per cell and incubated at 37 °C for 1 h. Infected cells were washed three times with PBS and incubated in medium containing gentamicin (100 μg/mL) for 2.5 h to kill extracellular bacteria. Gentamicin (100 μg/mL) was used to rapidly eliminate extracellular bacteria and prevent further infection by bacteria released from dead cells [87]. Aminoglycosides poorly penetrate the eukaryotic cell membrane when used in the incubation medium for short periods (usually less than 4 h) [88,89]. Cells were then washed three times with PBS and lysed using 0.5% Triton X-100 for 10 min. Appropriate dilutions of the lysates were spread on BHI agar. Three independent experiments were performed with four biological replicates for each infection.

### 4.7. Virulence in Mice

The virulence of *L. monocytogenes* strain F2365, mutant strains, and complemented strains were compared in a mouse infection model as previously described [86]. Sixty 8-week-old specific pathogen-free female BALB/c mice (Jackson Laboratory, Sacramento, CA, USA) were housed in 12 cages (5 mice/cage) according to treatment groups. Bacteria were harvested at an OD_600_ of ∼1.0 and washed with PBS. The bacterial density in overnight cultures was estimated from a previously prepared standard curve that correlated OD_600_ to viable-bacteria numbers. Average CFU was calculated based on serial dilutions and calculating the average colony numbers on agar plates. The OD_600_ was adjusted so that approximately 5 × 10^4^ CFU of each bacterial strain was inoculated intraperitoneally in each mouse in 100 μL of sterile PBS. Five mice were used for each strain. Mice were visually evaluated every two hours during the day for signs of illness or discomfort throughout the experiment. At 72 h post-infection, mice were euthanized followed by collection of liver and spleen samples. Tissue samples were weighed and homogenized in PBS. Homogenates were serially diluted and spread on BHI agar to determine numbers of viable bacteria.

### 4.8. Statistical Analysis

The data from adhesion, invasion, and in vivo virulence are presented as mean ± standard error (SE). The dot plots of bacterial numbers in each mouse were generated using GraphPad Prism 8 software, along with the median values. For in vivo experiment, a non-parametric Mann-Whitney test was used to detect the statistical significance in bacterial load in liver and spleen of infected mice between F2365 parent strain, mutants, and complemented strains. *p* values < 0.05 were considered statistically significant in all analyses. Statistical analysis of adhesion and invasion assays were performed as previously described [86]. Briefly, visual assessment of histograms using PROC UNIVARIATE in SAS for Windows v9.4 (SAS Institute Inc., Cary, NC, USA) indicated that the data was approximately normally distributed following log_10_ transformation. Pairwise comparison of means was conducted with Tukey’s test. Analysis of variance (ANOVA) was performed using PROC GLM in SAS for Windows v9.4 to assess significant differences.

## Figures and Tables

**Table 1:** Bacterial strains and plasmids used in this study.

Bacterial Strains, Plasmid	Description	Source/Reference
***E. coli***		
DH5α and Top10	Competent cells	Invitrogen (Carlsbad, CA, USA)
***L. monocytogenes***		
F2365	wild-type serotype 4b strain	[79]
F2365Δ*aguB*	F2365Δ*aguB mutant strain*	This study
F2365Δ*inlC2*	F2365Δ*inlC2 mutant strain*	This study
F2365Δ*eiic*	F2365Δ*eiic mutant strain*	This study
F2365Δ*tkt*	F2365Δ*tkt mutant strain*	This study
F2365Δ*bglG*	F2365Δ*bglG mutant strain*	This study
F2365Δ*aguB*::*aguB*	F2365Δ*aguB*::pPL2-*aguB* complemented strain	This study
F2365Δ*inlC2*::*inlC2*	F2365Δ*inlC2*::pPL2-*inlC2* complemented strain	This study
F2365Δ*eiic*::*eiic*	F2365Δ*eiic*::pPL2-*eiic* complemented strain	This study
F2365Δ*tkt*::*tkt*	F2365Δ*tkt*::pPL2-*tkt* complemented strain	This study
F2365Δ*bglG*::*bglG*	F2365Δ*bglG*::pPL2-*bglG* complemented strain	This study
**Plasmids**		
pHoss1	8995 bp, pMAD::*secY* antisense, Δ*bgaB*	[78]
pPL2	6123 bp, PSA *attPP,* chl^r^	[80]
*pLm*Δ*aguB*	pHoss1::Δ*aguB*	This study
*pLm*Δ*inlC2*	pHoss1::Δ*inlC2*	This study
*pLm*Δ*eiic*	pHoss1::Δ*eiic*	This study
*pLm*Δ*tkt*	pHoss1::Δ*tkt*	This study
*pLm*Δ*bglG*	pHoss1::Δ*bglG*	This study
pPl2-*aguB*	pPL2::*aguB*	This study
pPL2-*inlC2*	pPL2::*inlC2*	This study
pPL2*-eiic*	pPL2::*eiic*	This study
pPL2-*tkt*	pPL2::*tkt*	This study
pPL2-*bglG*	pPL2::*bglG*	This study

**Table 2:** Summarizes information on the mutant strains and deleted proteins function.

Mutants	Locus Tag	Encoded Protein	Function
F2365Δ*aguB*	LMOf2365_0045	Putrescine carbamoyltransferase	Catalyzes the formation of putrescine from carbamoyl-putrescine during agmatine degradation
F2365Δ*inlC2*	LMOf2365_0281	Internalin C2	Virulence, modulate host inflammation
F2365Δ*eiic*	LMOf2365_0661	Fructose-like permease EIIC subunit 2	Putative fructose-like permease EIIC subunit 2 phosphotransferase system (PTS) enzyme
F2365Δ*tkt*	LMOf2365_1054	Transketolase_C	Transketolase, C-terminal subunit, putative transketolase, N-terminal subunit
F2365Δ*bglG*	LMOf2365_2763	Transcription antiterminator, BglG family	Beta-glucoside operon family transcription antiterminator

**Table 3:** Primers used to generate and verify in-frame deletion strains.

Primers	Description	Sequence (5’→3’) ^b^	RE ^a^
AguB_F01	A	AA**GTCGAC**TCCGTTCCAGTAGTCGCTCTA	*Sal*I
AguB_R938	B	CGGAATCACCCTGTAACTCGT	
AguB_F833	C	ACGAGTTACAGGGTGATTCCGCGTTTTAGTTGTGGAATCTGC	
AguB_F01	D	AA**CCATGG**TTTCGCTGCATACATTGCTAC	*Nco*I
AguB_Seq		ATTGCGGAGTTGAAAGGCAAT	
InlC2_F02	A	AAGTCGACTTCATGGACCAAGCTACCAAT	*Sal*I
InlC2_R954	B	ACCCTTCTGTGCGAAAGATGT	
InlC2_F933	C	ACATCTTTCGCACAGAAGGGTGGCAATTAGCTTTTGGGTAGG	
InlC2_R02	D	AA**CCATGG**ATATTCGGGCTTGCATAAACA	*Nco*I
InlC2_seq		CGAATCAGAATAAACTGTTGC	
Eiic_F01	A	AA**GTCGAC**GCAAAAGTGACAACCCCACTA	*Sal*I
Eiic_R967	B	TGGACAAATTCTTCCTCTTCA	
Eiic_F964	C	TGAAGAGGAAGAATTTGTCCACGAGGAAGCAGATTGTGTCAT	
Eiic_F01	D	AA**CCATGG**CGCCATTATGCTCTTTCAAAC	*Nco*I
Eiic_Seq		TATGGTTGGTTCGATTGTAGG	
Tkt_F01	A	AA**GTCGAC**AATTGCCGTCTATCTGATCCA	*Sal*I
Tkt_R900	B	CCTTTCTCTATTCACCGCGTA	
Tkt_F900	C	TACGCGGTGAATAGAGAAAGGCCTTGGAGACGAAAGAAGATG	
Tkt_R01	D	ATA**CCATGG**CAATGTGTCTCCACAAGAACG	*Nco*I
Tkt_seq		GTTACTGGGTAAAGCGAGAGG	
BglG_F01	A	AA**GTCGAC**CCGGTTGCCCTATATTTTAGC	*Sal*I
BglG_R975	B	CGCTGTCAATGGGTTTTGTTA	
BglG_F936	C	TAACAAAACCCATTGACAGCGGCATTACCAGACTACGGTTTCA	
BglG_F01	D	AA**CCATGG**CTGCTTGGCTCATATTGGAAA	*Nco*I
BglG_Seq		GGGTATTATTGCTTGGATATGA	
AguB_Comp_F01		AAA**GAGCTC**ATGGTTGAGGTGATAGAAATGA	*Sac*I
AguB_Comp_R01		AAA**GTCGAC**ATCTTATAAGCCAGCGCCATT	*Sal*I
InlC2_Comp-F01		AAA**GAGCTC**AATGGTAGCTGCTATTCTCGGTA	*Sac*I
InlC2_Comp-R01		AAA**GTCGAC**CACTTTGATTGTTTTGCGGAG	*Sal*I
EiiC_Comp_F01		AAA**GAGCTC**GGAGGATAACTAAATGAGAACGCTTA	*Sac*I
Eiic_Comp_R01		AAA**GTCGAC**TCATCCTTTCTAAATGTCTTCAA	*Sal*I
Tkt_Comp-F01		AAA**GAGCTC**TACGCGGTGAATAGAGAAAGG	*Sac*I
Tkt_Comp-R01		AAA**GTCGAC**CTTCATCTTCTTTCGTCTCCAAGG	*Sal*I
BglG_Comp_F01		AAA**GAGCTC**TGGTGATTTGTTTGAGAATTGAG	*Sac*I
BglG_Comp_F01		AAA**GTCGAC**TCGCGGTAACAAGCCTATTAGT	*Sal*I

^a^ RE stands for restriction enzyme embedded to the 5’ end of the primer. ^b^ The bold sequences represent the restriction enzyme recognition sites added to primer A and D. Underlined sequences in primer C reflect reverse complemented primer B sequence.
